# Multifactorial pathogenesis of rheumatoid arthritis: interaction between inflammation, metabolic dysregulation, and tissue mechanics

**DOI:** 10.3389/fimmu.2026.1825523

**Published:** 2026-04-24

**Authors:** Yundong Xu, Qianqian Yang, Hongting Lu, Jian Zhang, Jin Xu, Xi Liu, Jian Wang, Zhaohu Xie, Zhaofu Li

**Affiliations:** 1 First Clinical Medical College, Yunnan University of Chinese Medicine, Kunming, Yunnan, China; 2 Joint Graduate School of Traditional Chinese Medicine of China, Suzhou, China

**Keywords:** inflammation, metabolic dysregulation, precision therapy, rheumatoid arthritis, tissue mechanics

## Abstract

Rheumatoid arthritis (RA) is a chronic systemic autoimmune disease characterized by persistent synovitis, progressive joint destruction, and diverse extra-articular complications. Increasing evidence indicates that RA pathogenesis is not driven by isolated inflammatory events, but by a tightly interconnected network involving immune dysregulation, metabolic reprogramming, and aberrant mechanotransduction. This review synthesizes recent advances in these three pathogenic dimensions and proposes an integrated framework for understanding RA as a systemic, self-reinforcing disease process. We highlight how inflammatory circuits, particularly the IL-6/STAT3 and TNF-α/NF-κB axes, interact with autoantibody- and neutrophil extracellular trap-mediated immune propagation beyond the synovium. We further discuss how glycolytic rewiring, succinate accumulation, and microbiota-derived metabolites amplify inflammatory signaling and tissue remodeling. In parallel, altered extracellular matrix stiffness and activation of the integrin-FAK-YAP/TAZ pathway sustain pathogenic fibroblast behavior through mechano-epigenetic coupling. Collectively, these pathways form a feed-forward loop that links local synovial inflammation with systemic organ involvement. A systems-level understanding of these interactions may provide a stronger foundation for biomarker-guided stratification and the development of multi-target, mechanism-based therapeutic strategies in RA.

## 1 Introduction

Rheumatoid arthritis (RA) is a chronic, systemic autoimmune disease characterized by persistent synovial inflammation that progressively leads to joint destruction and extra-articular manifestations. Its pathogenesis is highly complex and involves dynamic interactions among inflammation, autoimmunity, metabolism, and tissue mechanics. Among the most clinically significant comorbidities associated with RA are cardiovascular disease (CVD), interstitial lung disease (ILD), and osteoporosis (OP). Epidemiological studies have highlighted the extensive systemic burden imposed by RA. Data from the Chinese RA Registry Database reported a baseline prevalence of CVD of 2.2% (95% CI: 2.0-2.5) among RA patients (1). In addition, ILD, a severe and potentially life-threatening extra-articular manifestation of RA, has a pooled prevalence of 18.7% (95% CI: 15.8-21.6). Despite antifibrotic interventions, the five-year survival rate of patients with RA-associated ILD remains approximately 66% (2). Osteoporosis is also a common comorbidity in RA, and patients with RA have a higher risk of fractures than the general population. This risk may be further increased by prolonged corticosteroid use, which is associated with reduced bone mineral density (3). RA should therefore not be regarded solely as a localized synovial inflammatory disorder, but rather as a systemic disease driven by multiple interconnected pathogenic processes. This perspective also highlights the limitations of the traditional joint-focused approach in capturing and managing the multi-organ manifestations of RA.

Systemic pathology in RA arises from the interplay of genetic susceptibility, environmental exposure, aberrant immune activation, and downstream changes in metabolism and tissue behavior. Genome-wide association studies have identified multiple susceptibility loci, including HLA-DRB1 shared epitopes and PTPN22 variants, while environmental factors such as smoking and gut microbiome dysbiosis further contribute to the breakdown of immune tolerance (4–7). This process promotes the generation of anti-citrullinated protein antibodies (ACPA) and neutrophil extracellular traps (NETs), both of which are increasingly implicated not only in sustaining synovial inflammation but also in linking local immune responses to extra-articular and systemic manifestations (6, 8, 9). Beyond immune activation alone, accumulating evidence suggests that inflammatory signaling in RA is tightly coupled to metabolic reprogramming and altered mechanical cues within the synovial microenvironment. These reciprocal interactions help drive persistent inflammation, stromal activation, tissue remodeling, and ultimately multi-organ involvement. Such complexity also highlights the need for an integrated framework that connects immune, metabolic, and mechanical processes in RA. In this review, we discuss these interconnected dimensions of RA pathogenesis and examine how they contribute to persistent synovitis, tissue remodeling, and extra-articular manifestations. In RA, inflammatory signaling, metabolic reprogramming, and mechanical stress form a feed-forward circuit: inflammation drives stromal activation, matrix remodeling, and altered cellular metabolism; metabolic stress amplifies inflammatory and mechanoresponsive pathways; and abnormal tissue mechanics further stabilize fibroblast activation and chronic cytokine production. This reciprocal coupling provides a unifying framework for persistent synovitis, structural damage, and extra-articular organ involvement.

## 2 Systemic inflammatory mechanisms in RA

### 2.1 Crosstalk between IL-6/STAT3 and TNF-α/NF-κB signaling

#### 2.1.1 Molecular basis of IL-6/STAT3 and TNF-α/NF-κB synergy

The systemic inflammatory cascade in RA is driven in part by the functional crosstalk between the IL-6/STAT3 and TNF-α/NF-κB pathways, which together amplify inflammatory signaling at both the transcriptional and cellular levels (10, 11). One key mechanism involves the interaction between phosphorylated STAT3 and the NF-κB p65 subunit, leading to the formation of a nuclear transcriptional complex that enhances the expression of proinflammatory mediators such as IL-6 and CCL20 (12–15). This positive feedback loop contributes to the persistence of synovial inflammation and promotes continued recruitment of inflammatory cells. In parallel, TNF-α can further enhance STAT3 activity through ERK1/2-mediated phosphorylation at Ser727, thereby prolonging STAT3 nuclear retention and strengthening its transcriptional output (16). Through these reciprocal interactions, the IL-6/STAT3 and TNF-α/NF-κB axes function not as isolated pathways but as an integrated inflammatory module that sustains cytokine production, synovial activation, and broader immune dysregulation (17). This cross-pathway synergy provides an important mechanistic basis for persistent inflammation and systemic tissue damage in RA, while also creating conditions that favor metabolic adaptation and stromal activation within the synovial microenvironment.

#### 2.1.2 Epigenetic reinforcement of chronic inflammatory signaling

**Figure 1:**
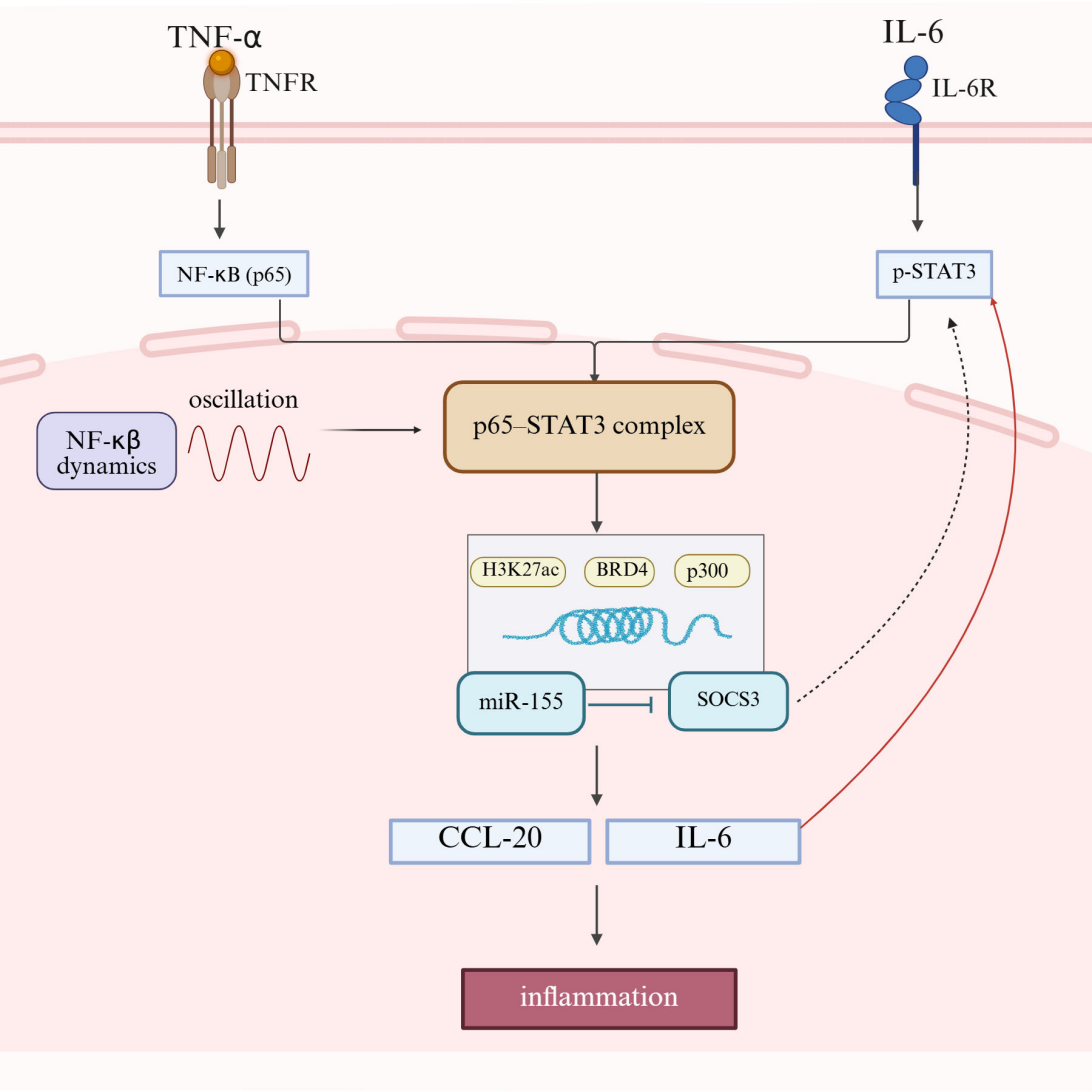
Crosstalk between TNF-α/NF-κB and IL-6/STAT3 signaling in rheumatoid arthritis. TNF-α activates NF-κB (p65) through TNFR, whereas IL-6 activates STAT3 through IL-6R. Convergence of these pathways promotes formation of the p65–STAT3 complex, which is associated with sustained inflammatory transcription. NF-κB dynamics, together with H3K27ac, BRD4, and p300, support persistent pathway activation, while the miR-155/SOCS3 loop further modulates signal amplification. Increased expression of downstream mediators such as IL-6 and CCL-20 contributes to chronic inflammatory activation in rheumatoid arthritis. Solid arrows indicate activation or progression; inhibitory bars indicate suppression.

Epigenetic regulation plays a critical role in stabilizing and amplifying chronic inflammatory signaling in RA, thereby extending the effects of cytokine-driven activation beyond transient signaling events. In RA synovial tissue, persistent IL-6 expression has been linked to the formation of an H3K27ac-marked super-enhancer in inflammatory macrophages, which strengthens chromatin accessibility and promotes sustained transcriptional activity (18, 19). Chromatin looping further facilitates physical interaction between this regulatory region and the IL-6 promoter, enhancing RNA polymerase II recruitment and reinforcing continuous IL-6 transcription (20). In parallel, STAT3 can cooperate with BRD4 and p300 to establish a permissive chromatin state at the TNF-α locus, thereby further amplifying inflammatory gene expression (19). In addition to chromatin remodeling, NF-κB-driven upregulation of miR-155-5p suppresses SOCS3, a negative regulator of STAT3 signaling, and thus reinforces the IL-6/STAT3 inflammatory circuit (21). Collectively, these epigenetic and post-transcriptional mechanisms do not merely accompany inflammation, but help lock inflammatory signaling into a self-sustaining state, thereby contributing to synovial persistence, tissue remodeling, and the broader metabolic and structural chronicity of RA (**Figure 1**).

#### 2.1.3 Dynamic encoding of NF-κB signaling

The temporal dynamics of NF-κB signaling are increasingly recognized as an important determinant of chronic inflammation in RA. Rather than acting as a simple on/off pathway, NF-κB can encode inflammatory signals through distinct oscillatory patterns, which influence chromatin accessibility, transcriptional persistence, and downstream tissue responses (22, 23). Under relatively weak or sustained TNF-α stimulation, low-frequency NF-κB nuclear translocation has been associated with prolonged accessibility at the promoters of inflammatory and matrix-remodeling genes, including IL-6 and matrix metalloproteinase-13 (MMP-13), thereby supporting persistent inflammatory transcription and synovial fibroblast activation (24). By contrast, stronger TNF-α stimulation or concurrent activation of the IL-6/STAT3 pathway may shift NF-κB activity toward a higher-frequency state, which has been linked to amplified inflammatory output, formation of transient transcriptional condensates, and inflammatory cell death programs (24–28). These dynamics may further enhance the release of mediators such as IL-1α and thereby extend inflammatory signaling beyond the synovium. In this context, NF-κB dynamic coding may contribute not only to local tissue destruction but also to the propagation of inflammatory injury across vascular and pulmonary compartments, thereby linking synovial inflammation to extra-articular disease progression, including RA-associated interstitial lung disease (29–32).

#### 2.1.4 Translational implications and therapeutic targeting

Mechanistic insights into the IL-6/STAT3 and TNF-α/NF-κB axes have created new opportunities for translational targeting in RA. In particular, strategies designed to simultaneously suppress convergent inflammatory pathways or disrupt their epigenetic reinforcement may offer advantages over single-node intervention, especially in patients with persistent synovial inflammation and progressive structural damage. Preclinical studies have shown that dual-pathway inhibition, including JAK- and BTK-related approaches, can attenuate joint swelling and bone destruction in experimental arthritis models (33). Likewise, BET inhibition has emerged as a potential strategy for dampening inflammatory transcription by interfering with BRD4-dependent enhancer activity and reducing IL-6/TNF-α-associated super-enhancer function (34, 35).

However, the translational significance of these approaches should be interpreted cautiously. Much of the current evidence remains preclinical, and whether these strategies can provide durable clinical benefit, improve extra-articular outcomes, or reduce comorbidity burden in RA remains uncertain, particularly because evidence for systemic benefit is substantially less developed than evidence for suppression of synovial inflammation. Combination strategies, including those aimed at modulating NF-κB dynamics or inflammatory cell death pathways, are mechanistically attractive, but their efficacy, safety, and patient-selection value require further validation (33). In addition, some proposed combination approaches for cardiovascular protection or systemic benefit need stronger disease-specific clinical evidence before their therapeutic relevance in RA can be established (36).

Taken together, current therapeutic development supports the concept that inflammatory signaling in RA is best understood as an interconnected network rather than a set of isolated pathways. From a translational perspective, future progress will likely depend on better integration of molecular stratification, extra-articular risk assessment, and mechanism-based combination therapy, rather than simply expanding the list of candidate drug targets.

Although IL-6/STAT3, TNF-α/NF-κB, and related inflammatory circuits are consistently implicated in RA progression, the relative dominance of these pathways varies across tissue settings and study models, and some mechanistic interactions remain better supported in experimental systems than in patient-based validation.

### 2.2 Autoimmune propagation beyond the synovium: ACPA, NETs, and cross-organ injury

### 2.2.1 ACPA-driven expansion of autoimmune injury beyond the joint

ACPA are central to the immunopathogenesis of RA and are widely regarded as key mediators of the loss of tolerance to citrullinated self-antigens (37). Beyond their established role in synovial inflammation, accumulating evidence suggests that the pathogenic relevance of ACPA may extend to extra-articular tissues. This broader effect is thought to arise not only from recognition of canonical synovial targets such as citrullinated vimentin, but also from cross-reactivity with citrullinated antigens detected in cardiovascular and pulmonary compartments, thereby supporting a model of autoimmune propagation beyond the joint (38, 39).

A mechanistic basis for this expanded autoreactivity lies in affinity maturation of autoreactive B-cell receptors. In RA, somatic hypermutation in antigen-experienced B-cell clones can increase both the affinity and breadth of ACPA binding to citrullinated epitopes, thereby facilitating recognition of structurally distinct self-antigens and reinforcing systemic autoreactivity (38). This feature is particularly important because it provides a plausible link between local breach of tolerance in the synovium and broader organ vulnerability, rather than treating extra-articular manifestations as unrelated downstream complications.

In the cardiovascular system, ACPA have been linked to endothelial injury and proatherogenic inflammation. Experimental studies suggest that binding of ACPA to citrullinated vascular antigens may promote complement deposition and enhance local inflammatory damage, while cross-reactivity with oxidized lipid-related structures may further support foam-cell formation and vascular lesion progression (39, 40). These mechanisms provide a biologically plausible explanation for the excess cardiovascular burden observed in RA, although direct causal attribution in patients remains difficult and the relative contribution of ACPA likely varies across vascular contexts.

ACPA-associated autoimmune injury may also extend to skeletal muscle, but the evidence in this area is more preliminary and mechanistically heterogeneous. Available studies suggest that ACPA may recognize citrullinated muscle-associated proteins, amplify NF-κB- and IL-6-related inflammatory signaling, and interfere with pathways involved in muscle maintenance, regeneration, and mitochondrial homeostasis (39, 41). These observations are consistent with a model in which autoantibody-driven inflammation converges with metabolic stress and impaired tissue repair to promote muscle wasting in RA, but direct evidence in human disease remains limited.

Taken together, current evidence supports an expanded view of ACPA as more than serological markers of RA. They may function as active mediators linking synovial autoimmunity to extra-articular tissue injury, particularly in cardiovascular and musculoskeletal systems. At the same time, the strength of evidence differs across organs, and careful distinction is still needed between mechanistic plausibility, experimental support, and clinically established organ-specific effects.

#### 2.2.2 Epigenetic remodeling mediated by NETs and cross-organ injury

Neutrophil extracellular traps (NETs) are increasingly recognized not only as antimicrobial structures, but also as active drivers of inflammatory amplification and epigenetic remodeling in RA and related autoimmune conditions (42). By releasing extracellular DNA, histones, and associated enzymes, NETs can reshape the local inflammatory microenvironment and influence chromatin regulation in surrounding stromal and immune cells. This broader view is important because it positions NETs not merely as byproducts of neutrophil activation, but as molecular platforms through which innate immune activation may be converted into persistent tissue injury and systemic pathological propagation.

In the pulmonary compartment, NET-associated epigenetic remodeling has emerged as a plausible mechanism linking chronic inflammation to fibrotic progression in RA-associated interstitial lung disease. Experimental studies suggest that NET formation is accompanied by chromatin decondensation and histone modification, while NET-related factors such as PADI4 and oxidative stress-responsive pathways may further reinforce profibrotic signaling (43–45). In this setting, NET-associated chromatin remodeling has been linked to increased expression of fibrotic mediators, including TGF-β1 and α-SMA, and to fibroblast activation, myofibroblast transition, and extracellular matrix deposition (46, 47). These findings support a model in which NETs connect immune activation with structural remodeling in the lung, although the relative contribution of specific epigenetic events in human RA-ILD remains incompletely defined.

NET-driven injury may also extend to the vascular compartment. Oxidized DNA species released during NET formation, including damage-associated nucleic acid signals, have been reported to activate endothelial stress pathways such as STING–IRF3 and RhoA/ROCK signaling, thereby impairing junctional organization and promoting proatherogenic endothelial dysfunction (48–53). This is mechanistically important because it provides a potential link between autoimmune inflammation and vascular barrier failure, lipid infiltration, and plaque progression. However, as with pulmonary fibrosis, much of the current evidence remains mechanistic or associative, and direct attribution of cardiovascular events in RA to NET-dependent pathways alone would be premature.

Emerging translational data further suggest that NET-related features may have cross-organ prognostic relevance. Recent single-cell and biomarker-based studies indicate that distinct neutrophil epigenetic states in synovial tissue and elevated circulating CitH3 levels may be associated with subsequent pulmonary or cardiovascular risk (54, 55). These observations are provocative because they raise the possibility that NET-associated signatures could eventually support patient stratification, but they should currently be interpreted as early-stage indicators rather than clinically established tools.

Overall, NETs appear to function as a mechanistic bridge between innate immune dysregulation, epigenetic remodeling, and organ-specific tissue injury. Their pathogenic significance in RA lies not only in amplifying local inflammation, but also in promoting fibrotic and vascular remodeling across compartments. This framework preserves the important insight that systemic complications in RA may arise from immune processes that are simultaneously inflammatory, epigenetic, and structural in nature.

#### 2.2.3 Translational targeting of the ACPA-NET axis

The emerging recognition of ACPA- and NET-driven pathology has opened new possibilities for mechanism-based intervention in RA, particularly in patients with extra-articular involvement or persistent inflammatory remodeling. From a translational perspective, this axis is attractive because it links autoantibody-mediated injury, innate immune amplification, and epigenetic dysregulation, thereby offering multiple therapeutic entry points rather than a single isolated target.

Among these, PAD4-related strategies are of particular interest because they directly intersect with NET formation and chromatin modification. Preclinical studies suggest that PAD4 inhibition can reduce CitH3 generation, attenuate profibrotic signaling, and partially reverse aberrant epigenetic states in fibroblasts, thereby limiting collagen accumulation and tissue remodeling (56–59). Likewise, histone-directed interventions such as HDAC inhibition have been reported to rebalance inflammatory chromatin states and suppress cytokine expression in synovial tissues (60–62). These approaches are mechanistically compelling because they do not simply block downstream inflammation, but attempt to interfere with the epigenetic reinforcement that stabilizes chronic immune injury.

B-cell- and autoantibody-directed strategies provide a complementary translational route. Targeting CD19^+^ B cells has recently shown preliminary clinical promise in isolated reports of treatment-refractory RA, supporting the broader concept that pathogenic autoantibody networks may be therapeutically modifiable (63). However, deeper depletion of autoreactive B-cell compartments may come at the cost of prolonged B-cell depletion/aplasia and other immune-related toxicities, as reported in the broader CD19 CAR-T autoimmune literature (64).

More experimental approaches, including nucleic acid delivery systems and combination regimens aimed at both autoantibody production and NET persistence, further illustrate the conceptual promise of this field (64–68). In particular, strategies designed to combine B-cell-directed therapy with degradation of NET-derived material are appealing because they address both upstream autoreactivity and downstream inflammatory scaffolding. Nevertheless, the current evidence for many of these approaches remains preclinical or early-phase, and robust validation in RA-specific extra-articular disease is still lacking.

Taken together, therapeutic development targeting the ACPA-NET axis highlights an important shift in RA research: from suppressing isolated inflammatory outputs toward disrupting the immune-epigenetic circuits that sustain systemic disease. The main challenge now is not the lack of plausible targets, but the need for stronger disease-specific clinical evidence, safer long-term modulation strategies, and better patient stratification to determine which individuals are most likely to benefit from such interventions.

**Figure 2:**
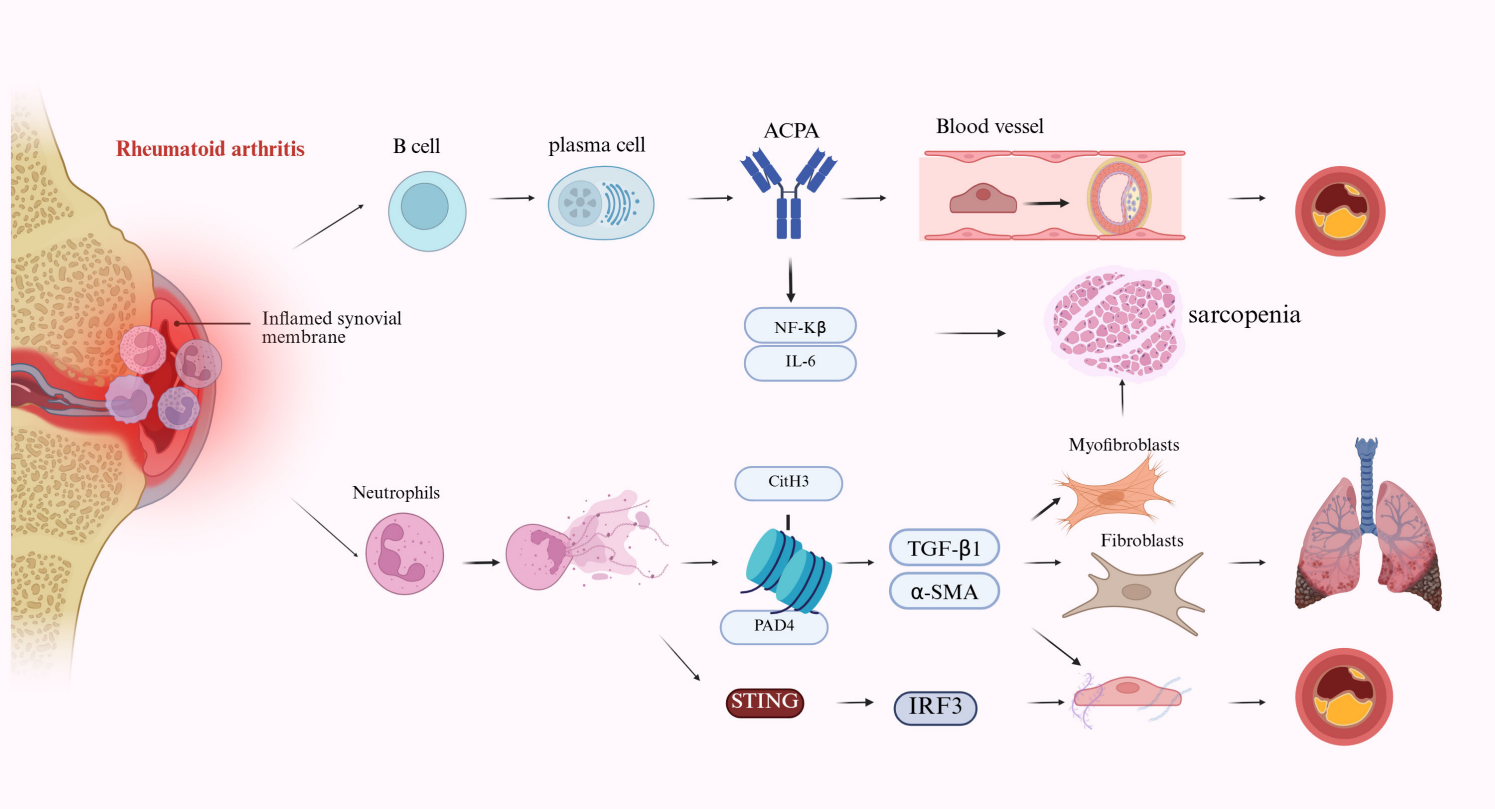
ACPA- and NET-associated extra-articular propagation in rheumatoid arthritis. Synovial inflammation promotes B-cell activation, plasma cell differentiation, and production of anti-citrullinated protein antibodies (ACPA). ACPA-associated signaling is linked to NF-κB- and IL-6-related inflammatory activation and may contribute to vascular injury and skeletal muscle dysfunction. In parallel, activated neutrophils release neutrophil extracellular traps (NETs), with PAD4 and CitH3 shown as representative NET-related components. NET-associated pathways are linked to TGF-β1/α-SMA-related fibroblast activation and fibrotic remodeling, as well as STING–IRF3-associated vascular injury. Together, these immune processes provide a framework for extension of rheumatoid arthritis beyond the joint to involve the vasculature, lung, and skeletal muscle. The pathways shown summarize proposed pathogenic relationships and do not imply equivalent levels of clinical validation across all organs.

However, the level of support is not uniform across organ systems: while several studies support strong associations between ACPA/NET-related immunity and extra-articular injury, direct causal and antigen-specific validation in RA patients remains incomplete in several proposed contexts (**Figure 2**).

### 2.3 Metabolic reprogramming axis: from energy stress to tissue remodeling in RA

#### 2.3.1 Metabolic reprogramming: dual driving of the Warburg effect and succinate accumulation

In RA, metabolic dysregulation is not merely a secondary consequence of inflammation, but a constitutive component of pathogenic tissue activation. A hallmark of this process is the upregulation of glycolysis in fibroblast-like synoviocytes (FLS), resembling the Warburg effect observed in tumor cells. Even in the presence of oxygen, activated FLS preferentially shift toward glycolytic energy production rather than full mitochondrial oxidative phosphorylation, thereby supporting rapid ATP generation, biosynthetic demand, and the persistent activation state of inflammatory and stromal cells. This metabolic reprogramming is accompanied by increased glucose uptake and contributes to the proliferation, invasiveness, and inflammatory persistence of synovial tissue (69, 70).

Succinate accumulation represents a second critical layer of metabolic rewiring in RA. As an intermediate of the tricarboxylic acid (TCA) cycle, elevated succinate levels are frequently associated with mitochondrial dysfunction and reduced succinate dehydrogenase (SDH) activity, and increased succinate concentrations have been detected in the synovial tissue of patients with RA compared with healthy controls (71). Importantly, succinate is not only a metabolic byproduct, but also an immunometabolic signaling molecule. In immune and stromal compartments, succinate can amplify inflammatory programs and promote the production of cytokines such as IL-1β and TNF-α, thereby linking mitochondrial metabolic stress to sustained inflammatory output (72).

Mechanistically, succinate exerts part of its pathogenic effect through succinate receptor 1 (SUCNR1)-dependent signaling. Upon binding to SUCNR1, succinate has been reported to activate the Gq-PLC pathway and to promote nuclear translocation of the Yes-associated protein/transcriptional co-activator with PDZ-binding motif (YAP/TAZ) transcriptional complex, thereby enhancing the transcriptional activity of synovial fibroblasts and reinforcing inflammatory activation (73, 74). Of particular interest, this pathway appears to intersect with integrin-FAK signaling, forming a self-reinforcing loop that links metabolic stress to mechanoresponsive transcription, synovial cell migration, and persistent tissue remodeling (75). This interaction is highly relevant to RA pathogenesis because it provides a mechanistic bridge between metabolic imbalance, inflammatory persistence, and altered tissue behavior, rather than treating these processes as isolated axes.

In addition to succinate, lactate accumulation and local lactate gradients also contribute to structural damage in RA. Increased lactate concentrations in the inflamed microenvironment have been shown to enhance the sensitivity of osteoclast precursor cells to receptor activator of nuclear factor κB ligand (RANKL), at least in part through monocarboxylate transporter 4 (MCT4), thereby promoting osteoclast activation and bone destruction (76). Thus, glycolytic rewiring in RA is not limited to fueling inflammation; it also remodels the biochemical microenvironment in ways that favor skeletal erosion.

Taken together, the Warburg-like shift, succinate accumulation, and lactate-rich inflammatory milieu define a coordinated metabolic program in RA. This program sustains synovial inflammation, amplifies mechano-inflammatory signaling, and promotes bone-destructive remodeling, highlighting that metabolic imbalance in RA is both an energetic crisis and a structural driver of disease progression.

#### 2.3.2 Microbiome-host interaction: TMAO-induced inflammation and microbiota dysbiosis

Metabolic imbalance in RA is shaped not only by intrinsic immune activation and synovial metabolic rewiring, but also by disruption of the gut microbiota-host metabolic axis. Increasing evidence suggests that alterations in intestinal microbial composition can influence systemic inflammatory tone, metabolite availability, and extra-articular risk. Among these changes, expansion of Prevotella-associated communities has been repeatedly reported in subsets of RA patients and has been linked to disturbed choline metabolism and elevated circulating levels of trimethylamine N-oxide (TMAO), a microbiota-derived metabolite with recognized pro-inflammatory and pro-atherogenic properties (77). Although the magnitude and consistency of microbiome alterations vary across cohorts, these observations support the concept that microbial dysbiosis contributes to the metabolic environment in which RA develops and persists.

TMAO is of particular mechanistic interest because it appears to act at the interface of vascular biology, immunity, and epigenetic regulation. Recent studies suggest that TMAO can exert dual regulatory effects through pathways involving Toll-like receptor 4 (TLR4)-phosphoinositide 3-kinase (PI3K)/protein kinase B (Akt) signaling. In the cardiovascular compartment, TMAO has been reported to enhance platelet activation, including GPIIb/IIIa-dependent responses, thereby favoring microthrombus formation within atherosclerotic lesions and increasing thrombo-inflammatory risk (78). In parallel, in adaptive immune cells, TMAO has been linked to inhibition of the methionine cycle in CD4^+^ T cells, leading to a reduced S-adenosylmethionine/S-adenosylhomocysteine (SAM/SAH) ratio, aberrant histone H3K4 methylation, enhanced T helper 17 (Th17) polarization, and increased IL-17A secretion (79). These findings are important because they suggest that a single microbiota-derived metabolite may simultaneously reshape vascular risk and immune polarization, thereby reinforcing the systemic character of RA. At the same time, the precise contribution of TMAO to RA-specific disease progression, as distinct from broader cardiometabolic inflammation, still requires careful interpretation.

In contrast to the pathogenic effects associated with TMAO, depletion of butyrate-producing microbiota highlights the loss of a potentially protective metabolic circuit. Reduced abundance of butyrate-producing taxa, particularly *Roseburia* spp., has been implicated in exacerbating metabolic disturbance and inflammatory severity in RA. Mechanistically, decreased butyrate availability has been associated with activation of histone deacetylase 6 (HDAC6), reduced H3K9ac density, suppression of the tumor suppressor gene cyclin-dependent kinase inhibitor 2A (CDKN2A), and increased expression of matrix-degrading mediators such as MMP-13 (80). This is a particularly valuable line of evidence because it links microbial metabolite depletion not only to immune dysregulation, but also to chromatin remodeling and tissue-destructive programs within the joint. In other words, dysbiosis in RA may operate through a double hit: generation of harmful metabolites such as TMAO and simultaneous loss of homeostatic metabolites such as butyrate.

**Figure 3:**
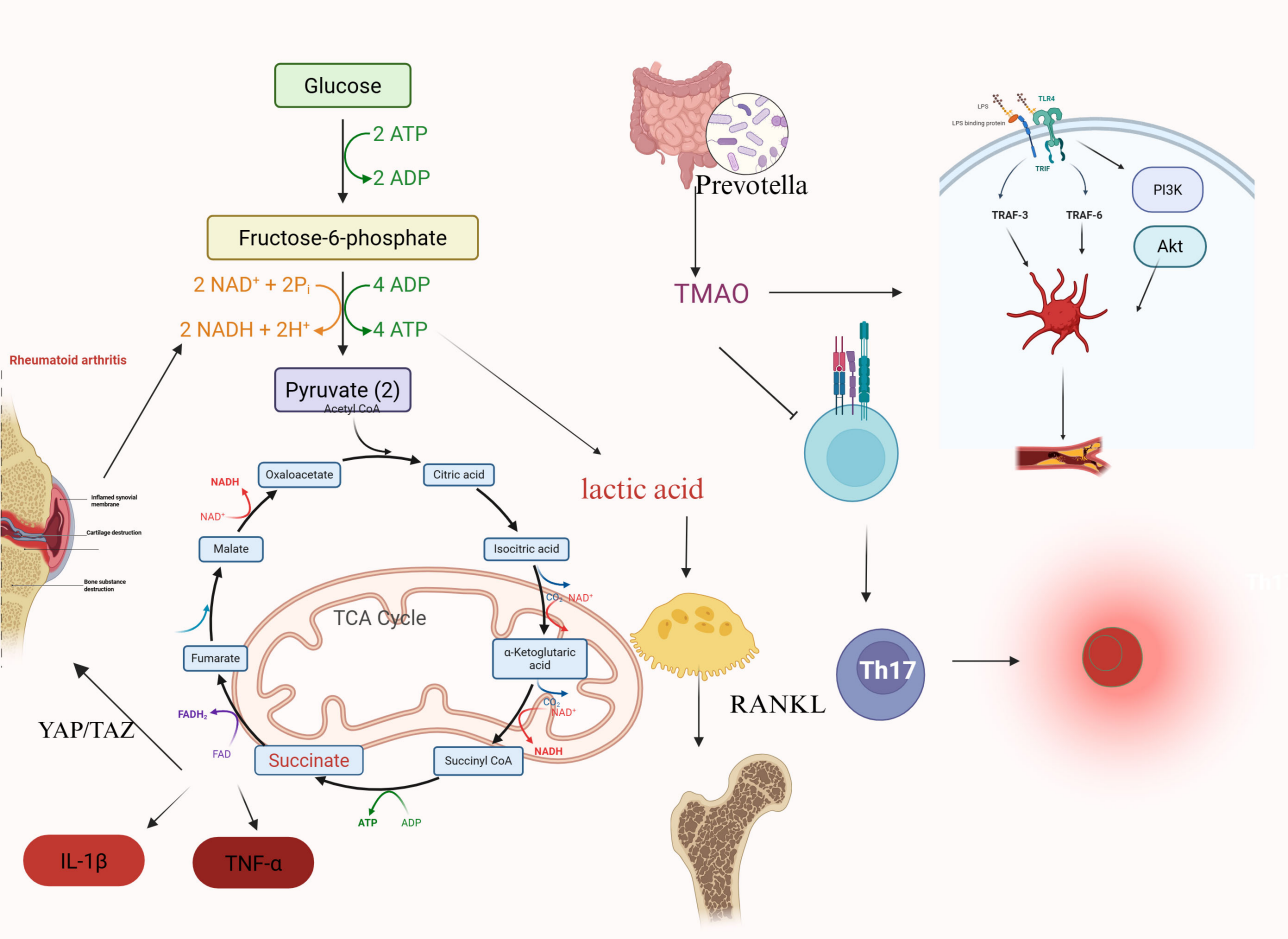
Integrin–FAK–YAP/TAZ signaling and epigenetic memory in rheumatoid arthritis synovial fibroblasts. Increased extracellular matrix (ECM) stiffness enhances integrin α5β1 engagement and activates focal adhesion kinase (FAK), coupled to cytoskeletal remodeling and downstream YAP/TAZ signaling. Nuclear YAP/TAZ activity is linked to H3K27ac, UHRF1, and DNMT, indicating coupling between mechanical cues, transcriptional activation, and epigenetic regulation. These events are associated with increased matrix gene expression, fibroblast invasion, and persistence of an activated stromal phenotype. Epigenetic memory further supports maintenance of a stiff ECM microenvironment, forming a feed-forward loop that sustains fibroblast activation and matrix remodeling in rheumatoid arthritis.

Overall, gut microbiota dysbiosis appears to contribute to a self-perpetuating cycle in RA. Elevated TMAO may potentiate immune activation, vascular inflammation, and thrombosis-related risk, whereas butyrate deficiency weakens immune-regulatory restraint and favors inflammatory and matrix-destructive signaling. Through these combined effects, disruption of the gut microbiota-host metabolic axis may accelerate not only articular destruction but also extra-articular manifestations, particularly in the cardiovascular and skeletal systems (81, 82). These observations underscore the growing importance of host-microbiome metabolism in explaining why RA behaves as a systemic disease rather than a joint-limited inflammatory disorder (**Figure 3**).

#### 2.3.3 Clinical strategy: interdisciplinary intervention targeting metabolic hubs

The recognition of metabolic imbalance as a core component of RA pathogenesis has stimulated growing interest in therapies that target metabolic hubs rather than solely suppressing downstream inflammatory mediators. This therapeutic perspective is especially attractive because metabolic interventions may simultaneously influence synovial activation, immune polarization, tissue remodeling, and extra-articular risk. However, as in other parts of RA translational research, it is essential to distinguish clearly between mechanistic promise, preclinical efficacy, and disease-specific clinical validation.

One emerging strategy involves repurposing metabolic regulators capable of modifying systemic energy handling. Empagliflozin, a sodium-glucose cotransporter 2 (SGLT2) inhibitor, has shown favorable effects on metabolic homeostasis in RA-related settings. By inhibiting renal glucose reabsorption, empagliflozin markedly increases urinary glucose excretion and improves glycemic control, with reported reductions in glucose variability and HbA1c (83). Beyond glucose lowering, it has also been associated with activation of AMPK signaling in skeletal muscle, increased phosphorylation activity, and restoration of fatty acid oxidation rates, suggesting broader reprogramming of systemic energy metabolism (84). These adaptations are mechanistically appealing because they may alleviate inflammatory stress and reduce the metabolic support for chronic synovial activation. Still, the extent to which such benefits reflect RA-specific immunometabolic modulation, rather than generalized improvement in metabolic status, remains an important question.

A second translational avenue focuses on restoration of microbiota-derived homeostatic metabolites. The combination of oral *Clostridium butyricum* supplementation with dietary fiber is noteworthy because it seeks not merely to suppress inflammation, but to rebuild a favorable metabolic niche in the intestine. Reported studies indicate that this strategy can increase intestinal butyrate concentration, inhibit HDAC6 activity, and restore H3K9ac density, changes that are consistent with improved immune regulation and attenuation of chronic inflammatory signaling (81). Conceptually, this is one of the more compelling approaches in the metabolic field because it links microbiota engineering to epigenetic rebalancing. However, variability in colonization efficiency, dietary responsiveness, microbiome heterogeneity, and durability of therapeutic effect remain major translational barriers.

A third strategy targets harmful microbial metabolites directly. TMAO has attracted considerable attention because of its established links to atherosclerosis and cardiovascular disease, which are highly relevant to RA comorbidity. Inhibitors of TMA production such as 3,3-dimethyl-1-butanol (DMB) have been shown to suppress the conversion of choline to trimethylamine and substantially reduce plasma TMAO levels (85). Experimental data further suggest that suppression of TMAO production may attenuate vascular inflammation, endothelial-immune dysregulation, and microthrombus formation within atherosclerotic plaques (86). These findings are promising because they address one of the major systemic consequences of RA, namely increased cardiovascular burden. Nevertheless, at present they are best regarded as proof-of-concept strategies rather than established RA therapies.

Taken together, metabolic intervention in RA should not be viewed as an alternative to immune modulation, but as a complementary strategy for interrupting the feed-forward loop between inflammation, energy stress, microbial dysbiosis, and tissue remodeling. The field is moving beyond the idea that metabolism merely reflects disease activity and toward the recognition that metabolic hubs themselves may be actionable determinants of RA progression. The key challenge now is to determine which interventions truly modify RA-specific pathogenic circuits, which patient subsets are most likely to benefit, and how metabolic targeting can be integrated rationally with existing anti-rheumatic therapies.

Collectively, these studies support a meaningful contribution of metabolic dysregulation to RA pathogenesis, but the reported metabolites do not yet carry equivalent mechanistic or clinical weight, and in several cases their roles remain closer to amplification or association than to fully established causal drivers.

### 2.4 Mechanical stress axis: epigenetic regulation induced by mechanical stress

#### 2.4.1 Mechanical signal transduction: epigenetic activation of the integrin-FAK-YAP/TAZ pathway

The synovial damage and persistent joint inflammation observed in rheumatoid arthritis (RA) are not driven solely by aberrant immune activation, but are also closely linked to abnormalities in mechanical signaling within the inflamed synovial microenvironment. During disease progression, fibroblast-like synoviocytes (FLS) are continuously exposed to altered biomechanical cues generated by synovial hyperplasia, extracellular matrix (ECM) remodeling, cartilage erosion, and abnormal joint loading (87). These cues are not passive byproducts of inflammation; rather, they are sensed and converted into intracellular signaling programs that reinforce inflammatory activation, stromal aggressiveness, and pathological tissue remodeling (88). In this context, mechanical stress includes forces transmitted to the cell membrane, focal adhesions, and cytoskeletal system by endogenous and exogenous stimuli, and changes in these cues can profoundly influence not only cellular behavior but also the epigenetic states that stabilize pathogenic phenotypes (89).

In RA synovial tissue, mechanical stress is primarily sensed through integrin receptors, particularly integrin α5β1, which function as key molecular interfaces between the ECM and intracellular Mechan transduction machinery. Once activated by increased substrate stiffness or abnormal tensile stress, integrin α5β1 transduces signals to focal adhesion kinase (FAK), thereby initiating phosphorylation-dependent downstream cascades that regulate cytoskeletal organization, cell survival, migration, and inflammatory gene expression (90, 91). Importantly, this pathway does not operate in isolation from inflammatory signaling. Rather, inflammation-driven ECM accumulation and tissue stiffening appear to enhance integrin engagement, while integrin-FAK activation in turn promotes the invasive and pro-inflammatory behavior of RA-FLS, establishing a feed-forward relationship between stromal remodeling and inflammatory persistence (92). Accordingly, activation of the integrin-FAK axis is increasingly regarded as a hallmark of aberrant mechanotransduction in RA and as an important bridge linking tissue mechanics to sustained synovial pathology (93).

A critical downstream consequence of FAK activation is the nuclear translocation and transcriptional activation of Yes-associated protein (YAP) and transcriptional co-activator with PDZ-binding motif (TAZ), two central mechanosensitive regulators that translate physical cues into durable transcriptional outputs. Under mechanically activated conditions, YAP/TAZ accumulate in the nucleus and cooperate with chromatin-modifying cofactors such as p300 to promote transcription of pro-inflammatory and matrix-remodeling genes, including IL-6 (94). This process is especially significant because it connects mechanotransduction directly to epigenetic regulation. Studies suggest that YAP/TAZ recruitment to H3K27ac-enriched regulatory regions enhances enhancer activity and supports the formation or stabilization of super-enhancer-like transcriptional states, thereby sustaining inflammatory gene expression beyond the initial mechanical trigger. In other words, mechanical stimuli in RA are not simply transient upstream activators; they may reprogram chromatin accessibility and transcriptional competence in ways that favor persistent inflammatory output (95).

This mechanotransduction program is also highly relevant to the broader integrative framework of RA pathogenesis because it interfaces with both inflammatory and metabolic pathways. On the inflammatory side, YAP/TAZ-dependent transcriptional amplification can reinforce IL-6-centered cytokine circuits already discussed in earlier sections, thereby strengthening reciprocal interactions between stromal cells and immune effectors. On the metabolic side, mechanically activated FLS exist within a microenvironment characterized by altered glycolysis, lactate accumulation, and mitochondrial stress, all of which may further support cytoskeletal remodeling and pathogenic transcriptional activation. Thus, the integrin-FAK-YAP/TAZ pathway should not be viewed as a purely biomechanical module, but rather as a convergence node at which inflammatory signals, metabolic adaptation, and tissue stiffness are integrated into a stable pathogenic program.

**Figure 4:**
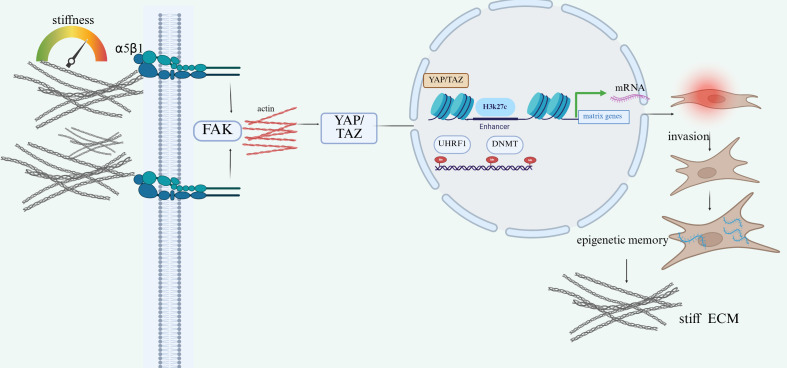
Metabolic reprogramming and microbiota-related signaling in rheumatoid arthritis. Enhanced glucose utilization and altered pyruvate/tricarboxylic acid (TCA) cycle metabolism are associated with accumulation of metabolites such as succinate and lactic acid, which contribute to inflammatory signaling and tissue remodeling in rheumatoid arthritis. Succinate is linked to downstream inflammatory mediators including IL-1β, TNF-α, and YAP/TAZ-related signaling, whereas lactic acid is associated with osteoclast-related tissue damage. In parallel, Prevotella-associated dysbiosis may increase trimethylamine N-oxide (TMAO), promoting immune activation, Th17 responses, RANKL-associated bone destruction, and vascular injury through TLR4–PI3K/Akt signaling. These pathways highlight the interaction between cellular metabolism, microbiota-derived metabolites, immune activation, and structural damage in rheumatoid arthritis.

An especially important implication of this pathway is that mechanically induced changes in RA-FLS may persist even after the initiating stimulus has been attenuated. In this setting, the concept is better described as persistent epigenetic memory rather than “intergenerational transmission,” because the available evidence more convincingly supports durable maintenance of an activated chromatin state within pathogenic stromal cells than true transgenerational inheritance. In RA-FLS, sustained accessibility of inflammatory loci, including the IL-6 promoter region, may remain elevated after removal of mechanical stress, suggesting that mechanotransduction can leave a durable epigenetic imprint (96). Molecules involved in epigenetic maintenance, including ubiquitin-like with PHD and RING finger domains 1 (UHRF1), may participate in this process by supporting DNA methylation maintenance and reinforcing DNA methyltransferase (DNMT)-associated chromatin memory programs. Through such self-reinforcing epigenetic regulation, repetitive mechanical stress may lock FLS into a chronically activated phenotype that continues to drive synovial inflammation, invasive behavior, and joint destruction even when the initial biomechanical input fluctuates (97) (**Figure 4**).

#### 2.4.2 Multifunctional effects of mechanical stress

The pathological significance of mechanical stress in RA extends well beyond local signal transduction in synovial fibroblasts. Once established, abnormal mechanical loading may influence tissue remodeling across multiple organ systems through coupled biomechanical, inflammatory, and molecular mechanisms. This broader perspective is important because it helps explain why RA cannot be understood simply as a joint-confined inflammatory disease. Instead, mechanical dysregulation appears to function both as a local amplifier of synovial pathology and as a contributor to systemic complications, although the strength of evidence varies considerably across tissues and should be distinguished accordingly (98).

One relatively well-supported consequence of mechanical dysregulation in RA involves the skeletal system. Physiological mechanical loading is essential for maintaining bone homeostasis, in part through activation of osteoblastic pathways such as Wnt/β-catenin signaling. When loading is chronically reduced, as may occur with severe joint pain, prolonged immobility, reduced physical activity, or inflammation-associated functional decline, bone-forming signals are suppressed and osteogenic capacity decreases. Experimental observations indicate that marked reduction in biomechanical stimulation can impair osteoblast activity, reduce bone formation, and accelerate loss of bone mineral density (99). At the same time, mechanical insufficiency may also enhance osteoclast-mediated resorption, thereby intensifying skeletal degradation. In RA, these effects likely interact with inflammatory osteoimmunological mechanisms rather than replacing them, meaning that mechanical deficiency should be interpreted as a co-driver that exacerbates osteoporosis risk in an already pro-resorptive inflammatory setting (100).

Mechanical stress also participates in shaping the structural and functional properties of the synovial niche itself. In RA, chronic inflammation drives extensive remodeling of synovial tissue, including activation and expansion of RA-FLS, enhanced synthesis of ECM components, and progressive distortion of normal tissue architecture (101, 102). Increased deposition of fibronectin, type I collagen, and other matrix components contributes to the emergence of a fibrotic-like synovial environment. This remodeled matrix is not merely a structural consequence of inflammation; it is likely to alter local tissue stiffness and thereby modify the mechanical landscape perceived by resident cells (103). Although direct *in vivo* quantification of synovial stiffness in RA remains limited, *in vitro* and ex vivo studies using atomic force microscopy and stiffness-controlled substrates consistently show that RA synovial cells respond strongly to rigid microenvironments. Under increased stiffness, RA-FLS display enhanced migration, invasiveness, and inflammatory activation, implying that inflammation-induced matrix remodeling generates a secondary biomechanical signal that further drives pathology. This creates a mechanobiological feedback loop in which inflammation alters tissue mechanics, altered tissue mechanics amplify FLS pathogenicity, and pathogenic FLS then further remodel the matrix (104, 105).

This feedback architecture is particularly important because it provides one of the clearest mechanistic connections among the three major themes of this review. Inflammatory cytokines promote synovial remodeling; metabolic changes support the high biosynthetic and contractile demands of activated stromal cells; and the resulting mechanical stiffening feeds back through integrin-FAK-YAP/TAZ signaling to reinforce inflammatory and invasive programs. Thus, tissue mechanics should not be treated as a third parallel axis alongside inflammation and metabolism, but as a force-dependent layer of regulation embedded within both.

The possibility that mechanical dysregulation in RA may also contribute to extra-articular pathology, particularly in the lung, is biologically compelling but supported by a more indirect evidence base. In advanced RA, chronic structural damage can involve thoracic joint components such as the sternoclavicular and costovertebral articulations, potentially altering chest wall biomechanics and thereby affecting respiratory mechanics. On this basis, it has been proposed that altered thoracic loading may influence the pulmonary microenvironment, including stress transmission to alveolar regions (106–108). However, this interorgan connection should be framed carefully. Direct evidence linking altered synovial mechanics in RA to alveolar dysfunction remains limited, and the current literature supports plausibility more strongly than direct causality.

What is better established is that alveolar epithelial cells, particularly alveolar type II cells, are highly mechanosensitive and respond to abnormal stretch and substrate stiffness through activation of Hippo-YAP/TAZ signaling. Under such conditions, these cells can undergo epithelial-mesenchymal transition (EMT)-associated changes, including downregulation of E-cadherin and upregulation of Vimentin and ZEB1, together with increased migratory behavior and enhanced expression of profibrotic genes (109, 110). These findings are relevant to RA because they suggest that once the pulmonary microenvironment is mechanically perturbed, mechanically activated transcriptional programs may promote fibrotic remodeling in a manner conceptually analogous to stiffness-driven activation in the synovium.

The mechanistic relevance becomes stronger when YAP/TAZ signaling is considered together with TGF-β1, a central fibrotic mediator in RA-associated interstitial lung disease (RA-ILD). YAP can enhance TGF-β1 expression and cooperate with Smad-dependent transcriptional machinery to activate fibroblast-associated gene programs that promote excessive ECM deposition and fibrosis progression (111). From a systems perspective, this suggests that inflammatory joint disease, matrix remodeling, altered tissue mechanics, and fibrotic signaling may be linked through shared mechanosensitive transcriptional regulators (112). Nevertheless, the degree of direct inter-organ coupling remains unresolved. It is therefore most defensible to state that current evidence supports a biologically plausible framework connecting mechanical dysregulation to pulmonary fibrotic remodeling in RA, rather than a fully established linear pathway from synovial stiffness to RA-ILD.

This distinction matters for a review article of this type. A stronger manuscript does not overclaim; it shows where the evidence is robust, where it is suggestive, and where mechanistic extrapolation remains necessary. In the mechanical stress axis, the strongest evidence currently supports local stiffness-dependent activation of RA-FLS and bone-related consequences of altered loading, whereas the cross-organ pulmonary extension remains an important but still partially inferential component of the model. Framing the literature in this way increases, rather than decreases, the intellectual strength of the section.

#### 2.4.3 Clinical strategies for mechanical intervention

The growing recognition of mechanical dysregulation as a pathogenic component of RA has generated increasing interest in therapeutic strategies that target mechanobiological processes. However, the translational significance of this field depends not on listing isolated technologies or candidate targets, but on clarifying how mechanistic insight can be leveraged clinically and where the current boundaries of evidence lie. In this sense, clinical translation in the mechanical domain should be understood as an effort to interrupt the feed-forward loop linking inflammation, stromal remodeling, altered tissue stiffness, and persistent pathogenic activation.

One emerging strategy is the use of computational precision models, including digital twin frameworks, to integrate multidimensional data relevant to mechanobiological disease states. By combining single-cell mechanogenomic profiles, joint kinematics, imaging-derived structural information, and circulating metabolic or inflammatory biomarkers, such systems may help reconstruct patient-specific disease dynamics and identify dominant regulatory nodes (113). In principle, this could allow better stratification of patients in whom mechanically reinforced inflammatory programs are especially prominent. The appeal of this approach lies in its ability to convert complex, cross-scale biological information into clinically actionable predictions (114, 115). However, despite its conceptual sophistication, digital twin technology remains an emerging translational platform rather than an established clinical tool in RA. Its predictive value, standardization, and real-world decision-making utility still require substantial validation.

A second translational direction involves the development of mechanoresponsive biomaterials for targeted drug delivery within diseased joints. Smart materials such as hydrogel microspheres can be engineered to respond to features of the pathological synovial niche, including elevated reactive oxygen species (ROS), acidic pH, and abnormal mechanical overload (116). In theory, these systems allow drugs to accumulate preferentially in mechanically and biochemically abnormal regions, thereby improving local retention, enhancing therapeutic concentration at the site of injury, and reducing off-target exposure (117). This is particularly attractive in RA because the synovium represents a microenvironment in which inflammatory and mechanical abnormalities are spatially concentrated (118). Preclinical studies have shown encouraging results, especially in cartilage-protective settings and local tissue repair models (119). Nevertheless, these technologies remain largely at the proof-of-concept or early translational stage, and their long-term performance, manufacturability, and disease-specific efficacy in human RA remain to be established.

Among currently proposed approaches, direct targeting of early mechanotransduction in RA-FLS is one of the most mechanistically mature. In rheumatoid synovial tissue, RA-FLS predominantly sense ECM alterations through integrin α5β1 and transmit these cues via FAK, making this pathway a critical entry point for interrupting stiffness-dependent pathogenic activation (120). Experimental studies demonstrate that RA-FLS cultured on substrates with increased stiffness show greater FAK phosphorylation, enhanced migratory and invasive behavior, and upregulated expression of pro-inflammatory mediators such as IL-6 and MMP3 (121). These findings make FAK a particularly attractive target because it lies upstream of multiple downstream consequences, including cytoskeletal reorganization, inflammatory transcription, and YAP/TAZ activation. Small-molecule inhibitors such as PF-573228 have been shown to suppress FLS mechanosensing and attenuate abnormal migration and inflammatory activation, suggesting that pharmacological blockade of the integrin-FAK axis could disrupt a central amplification mechanism in the mechanically remodeled synovium (122, 123).

Importantly, the value of such interventions is not merely that they add another molecular target to the therapeutic landscape. Rather, they reflect a conceptual shift in RA treatment strategy: from suppressing isolated inflammatory mediators to dismantling the tissue-contextual circuits that stabilize chronic disease. Mechanical intervention is especially relevant in this regard because it addresses a layer of pathogenic reinforcement that is not fully captured by conventional cytokine-centered frameworks. When the synovial matrix becomes progressively stiff, when stromal cells acquire mechanically imprinted inflammatory phenotypes, and when biomechanical signals are converted into durable epigenetic programs, immune suppression alone may not fully reverse the disease state. This is precisely where mechanobiology-informed therapy may provide complementary value.

At the same time, a critical perspective is essential. Not all proposed mechanical interventions are supported by the same level of evidence. FAK-centered approaches currently have relatively strong mechanistic rationale but remain largely preclinical. Mechanoresponsive biomaterials are technologically promising, yet their clinical scalability and disease specificity remain unresolved. Digital twin systems may eventually support precision medicine, but at present they should be presented as forward-looking integrative tools rather than validated therapeutic decision engines. A strong translational discussion therefore requires explicit distinction between mechanistic plausibility, experimental efficacy, and clinical readiness.

Taken together, the clinical significance of the mechanical stress axis lies not in replacing established immune-targeted therapies, but in expanding the therapeutic logic of RA. The most compelling future direction is likely to involve combination strategies in which anti-inflammatory treatment is integrated with interventions that modify pathogenic tissue mechanics, stromal mechanosensing, or niche-specific drug delivery. Such an approach is more consistent with the systems-level pathogenesis of RA described throughout this review and better reflects the reality that chronic disease persistence arises from reciprocal reinforcement among inflammatory, metabolic, and mechanical circuits.

Compared with the relatively well-supported evidence for stiffness-dependent activation of synovial fibroblasts in the local joint microenvironment, the extension of mechanotransduction-driven remodeling to extra-articular organs remains more inferential and still requires stronger RA-specific validation.

### 2.5 Integrated model of RA pathogenesis

Taken together, the evidence discussed above supports an integrated model of RA in which inflammation, metabolic dysregulation, and tissue mechanics are mutually reinforcing rather than independent processes. Inflammatory activation drives metabolic rewiring and matrix remodeling; metabolic stress enhances inflammatory and mechanoresponsive signaling; and altered tissue stiffness stabilizes pathogenic fibroblast behavior and chronic cytokine production. Once established, this self-sustaining loop may support disease progression even when the initiating trigger is no longer dominant, and may also help explain the extension of RA pathology beyond the joint.

## 3 Technological innovation and mechanism-based precision intervention in RA

Modern medicine is undergoing a significant transformation in the management of complex diseases such as RA, driven by the convergence of technological innovation and personalized therapeutic strategies. However, in RA, the value of emerging technologies lies not merely in their novelty, but in their ability to translate the integrated biology of immune dysregulation, metabolic disturbance, and biomechanical remodeling into more precise and stratified interventions. In this context, multimodal targeted delivery systems, gene-editing approaches, mechanobiology-informed devices, and high-resolution molecular profiling collectively provide a framework for overcoming the limitations of conventional treatment modalities.

In the field of multimodal targeted delivery, intelligent delivery systems such as responsive hydrogel microspheres can dynamically regulate drug release according to pathological microenvironmental cues, including hypoxia, excessive ROS production, and metabolic imbalance. Such systems offer the dual advantage of improving targeting precision and reducing systemic adverse effects. Recent studies have shown that succinate-ROS-responsive hydrogel microspheres represent a promising therapeutic strategy for arthritis, as they can attenuate cartilage degradation, suppress inflammatory responses, and enhance intra-articular drug retention, thereby improving therapeutic efficacy (124, 125). Nevertheless, these systems should currently be regarded as preclinical or early translational platforms rather than mature clinical therapies for RA.

Gene-editing technology, particularly the CRISPR/Cas9 system, has also demonstrated substantial potential in the treatment of bone and joint diseases. By enabling precise modification of target genes, CRISPR/Cas9 offers the possibility of intervening at the level of core pathogenic mechanisms rather than merely suppressing downstream inflammatory consequences. For example, CRISPR/Cas9-mediated upregulation of *FGF18* in chondrocytes has been shown to induce cartilage regeneration, attenuate inflammatory responses, and prevent extracellular matrix degradation, thereby slowing the progression of arthritis (126, 127). These findings provide not only new insight into articular cartilage repair but also a conceptual basis for future personalized therapies. However, in RA, the clinical translation of gene editing remains limited by challenges including delivery specificity, off-target effects, long-term safety, and uncertain disease-context applicability. Therefore, it should be presented as a promising but still exploratory strategy rather than an imminent therapeutic solution.

Significant advances have also emerged in mechanobiology-informed intervention strategies for joint disorders. Conventional orthotic devices and assistive technologies are often constrained by their limited adaptability and inability to modulate mechanical loading with precision. Wearable sensors and AI-assisted analytics may help monitor joint-related functional changes and detect RA flares in real time, and could eventually inform more adaptive assistive or orthotic strategies, although evidence for disease-modifying benefit remains limited.

More recently, the integration of wearable biosensors and AI-assisted algorithms has created the possibility of dynamic optimization of mechanical intervention. By continuously monitoring joint stress in real time, these systems may allow autonomous adjustment of orthotic pressure and thus enable more precise and adaptive regulation of joint loading conditions (128). Conceptually, this is particularly relevant in RA, where abnormal mechanical stress does not merely accompany inflammation but actively contributes to synovial activation, matrix remodeling, and disease persistence. Still, the therapeutic value of these approaches in RA should be interpreted cautiously, as robust evidence for their disease-modifying efficacy remains limited.

Recent advances in precision medicine have further accelerated the development of individualized therapeutic strategies. Single-cell and spatial transcriptomic technologies, particularly high-resolution platforms such as 10x Genomics Genomics Visium HD, are reshaping the understanding of RA pathogenesis by enabling high-resolution characterization of cellular heterogeneity within synovial tissue. These technologies allow spatial mapping of immune cell subsets, fibroblast-like synoviocytes, and their associated transcriptional programs, thereby refining the molecular architecture of inflammatory lesions (129). Moreover, spatial transcriptomics enables investigation of key signaling pathways, including YAP/TAZ, together with their spatial relationships to epigenetic regulators such as H3K27ac, thus providing important molecular coordinates for precision-targeted intervention in RA (130).

These technological advances are valuable not simply because they generate more data, but because they help uncover how immunometabolic interactions and biomechanical cues converge during RA progression. In particular, they improve current understanding of early disease evolution and of how mechanical stimuli influence synovial cell behavior within specific pathological niches. Looking forward, the integration of digital twin technology may further strengthen personalized therapeutic frameworks. By incorporating patient-specific genomic, epigenomic, spatial, metabolic, and biomechanical information, digital twin models could theoretically simulate disease trajectories and support individualized treatment design. Such an approach may eventually shift RA management from conventional symptom control toward mechanism-based precision intervention through dynamic monitoring and adjustment of immune, metabolic, and joint functional states.

Taken together, these emerging technologies should not be viewed as isolated innovations, but as components of a broader translational framework for precision medicine in RA. Their real significance lies in their potential to connect mechanistic insight with stratified intervention, thereby advancing RA treatment beyond generalized immunosuppression toward more biologically informed and patient-specific therapeutic strategies.

## 4 Conclusion

This review supports a systems-level view of RA in which inflammatory, metabolic, and mechanical pathways form a reciprocal pathogenic network rather than separate disease modules. The evidence discussed here emphasizes the reciprocal interactions among immune imbalance, metabolic reprogramming, and biomechanical signaling, which together sustain synovial inflammation, promote tissue destruction, and contribute to extra-articular complications. Processes such as ACPA production and NET formation extend the consequences of immune dysregulation beyond the joint, while metabolic alterations, including enhanced glycolysis and the accumulation of pro-inflammatory metabolites such as succinate, further amplify inflammatory activation. At the same time, aberrant mechanotransduction promotes synovial fibroblast activation, matrix remodeling, and progressive structural damage.

These observations underscore why therapeutic strategies focused on single pathways often fail to fully address disease heterogeneity and systemic involvement in RA. Future research should further clarify how these interconnected mechanisms vary across disease stages, tissue contexts, and patient subsets, and should prioritize biomarkers and therapeutic strategies that reflect combined inflammatory, metabolic, and mechanical states. A more integrated framework may provide a useful basis for biomarker-guided stratification and mechanism-based combination therapy in RA.
